# Correlation between excessive sugar intake and overactive bladder in children

**DOI:** 10.3389/fped.2025.1581852

**Published:** 2025-05-08

**Authors:** Xu Cui, Bo-Chao Wei, Zhi-Qiang Chen, Long-Yao Xu, Bing-Qian Yin, Chao-Ming Zhou

**Affiliations:** ^1^ College of Clinical Medicine for Obstetrics & Gynecology and Pediatrics, Fujian Medical University, Fuzhou, China; ^2^ Department of Pediatric Surgery, Fujian Children’s Hospital (Fujian Branch of Shanghai Children’s Medical Center), College of Clinical Medicine for Obstetrics & Gynecology and Pediatrics, Fujian Medical University, Fuzhou, China; ^3^ Fujian Maternity and Child Health Hospital College of Clinical Medicine for Obstetrics & Gynecology and Pediatrics, Fujian Medical University, Fuzhou, China

**Keywords:** overactive bladder, child, OABSS, sugar intake, types of sugary foods

## Abstract

**Purpose:**

The aim of this study was to explore the relationship between excessive sugar intake and symptoms of paediatric overactive bladder (OAB).

**Methods:**

Clinical data was collected from 97 patients diagnosed with OAB at our hospital between July 2024 and January 2025. The relationships between sugar intake and OAB symptom scores were analysed.

**Results:**

A total of 97 children diagnosed with OAB were included in this study, with 78 (80.41%) exhibiting excessive sugar intake and 19 (19.59%) exhibiting non-excessive sugar intake. Patients in the excessive sugar intake group had a significantly longer duration of OAB symptoms compared to those in the non-excessive sugar intake group (*P* < 0.05). A comparison of OAB symptoms between the two groups revealed that patients in the excessive sugar intake group had significantly higher total scores on the overactive bladder symptom score (OABSS) and higher scores for daytime urinary frequency, urgency frequency, and urgency urinary incontinence compared to those in the non-excessive sugar intake group (*P* < 0.05). Correlation analysis between sugar intake and OAB symptoms revealed significant positive correlations between average daily total sugar intake and the symptom duration of OAB, OABSS total score, daytime urination frequency score, urgency frequency score, and urgency urinary incontinence score (*P* < 0.05). The duration of excessive sugar intake was significantly positively correlated with the symptom duration of OAB, OABSS total score, and urgency urinary incontinence score (*P* < 0.05). The average daily sugar intake from fruits was also significantly positively correlated with the symptom duration of OAB, OABSS total score, daytime urination frequency score, urgency frequency score, and urgency urinary incontinence score (*P* < 0.05).

**Conclusion:**

Excessive sugar intake was closely associated with the severity of OAB symptoms in children. Both the quantity of sugar intake and the duration of excessive sugar intake were positively correlated with the severity of OAB symptoms. Among all types of sugary foods consumed, the average daily intake of fructose was positively correlated with the severity of OAB symptoms.

## Introduction

Overactive bladder (OAB) is an extremely common functional bladder disorder during childhood. According to the definition of the International Children's Continence Society, OAB is characterized by urgency, increased urinary frequency, and nocturnal enuresis, and it may or may not be associated with daytime incontinence in the absence of urinary tract infection or other identifiable neurogenic pathology (1). The persistent and recurrent nature of this condition, coupled with the distressing experience of uncontrollable urgency and wetting accidents, significantly impairs the quality of life of affected children (2). In recent years, the prevalence of OAB in children has been increasing, with reported rates ranging from 5% to 12% (3, 4).

The pathogenesis of paediatric OAB remains unclear. The latest expert consensus on paediatric OAB suggests that the condition may be associated with various factors, including: improper toileting habits, obesity, and hormonal metabolic imbalances (5, 6). Additionally, recent studies have indicated that changes in lifestyle and dietary habits, particularly the increased consumption of sugary beverages, are significant contributors to the development of OAB in adults (7). However, research examining the correlation between excessive sugar intake and OAB in children remains limited both domestically and internationally. Our team has proposed the scientific hypothesis that excessive sugar intake is closely associated with symptoms of paediatric OAB. The aim of this study was to explore the relationship between excessive sugar intake and symptoms of paediatric OAB by analysing the associations between sugar intake and objective measures of OAB symptom scores.

## Methods

Clinical data were collected from 97 patients diagnosed with OAB at our hospital between July 2024 and January 2025. The data included age, sex, body mass index (BMI), duration of OAB symptoms, age at which diapers were discontinued, sugar intake (including total sugar intake, duration of excessive sugar intake, and daily sugar intake from various sugary foods), OAB symptom scores, maximum urinary flow rate, and average urinary flow rate. The non-invasive urine flow rate was measured via urodynamic detection equipment (Laborie, China), and the maximum and average urine flow rates were recorded. During testing, bladder volume was required to reach at least 50% of the child's expected bladder capacity.

The inclusion criteria were as follows: (1) children diagnosed with OAB and (2) aged between 4 and 18 years. The exclusion criteria were as follows: (1) inability to cooperate with the study, (2) current urinary tract infection, (3) neurogenic bladder, (4) history of urethral injury or surgery, (5) lower urinary tract malformation, and (6) refusal to participate in the study.

The diagnostic criteria for OAB include the following: (1) daytime frequency of urination exceeding 12 times per day, (2) presence of symptoms, including urgent urinary incontinence and nocturnal enuresis, (3) postvoid residual examination showing less than 20 ml of residual urine in the bladder, and (4) absence of other neurological bladder diseases and no evidence of urinary tract infection (8).

The overactive bladder symptom score (OABSS) was used to collectively express OAB symptoms (9). The score consists of 4 questions: (1) “How many times do you typically urinate from waking in the morning until sleeping at night?” (2) “How many times do you typically wake up to urinate from sleeping at night until waking in the morning?” (3) “How often do you have a sudden desire to urinate, which is difﬁcult to defer?” (4) “How often do you leak urine because you cannot defer the sudden desire to urinate?” A higher score corresponds to a greater severity of symptoms. OABSS ≤5 was classified as mild, 6≤ OABSS ≤11 was classified as moderate, and OABSS ≥12 was classified as severe.

The criterion for excessive sugar intake was based on the recommendations of the Dietary Guidelines for Chinese Residents (10). Excessive sugar intake in children was defined as an average daily sugar intake of ≥50 g. To categorize the types of sugar intake, we recorded the most frequently consumed sugary foods in the week preceding the clinic visit. These were classified into the following categories: carbonated beverages, fruit juices (including apple, orange, grape, and watermelon juices), fruits (including apples, grapes, oranges, and watermelons), other sugary drinks (e.g., yogurt-based drinks), sugary tea beverages, chocolate, and ice cream. To accurately calculate the average daily sugar intake of the children, we adopted the methodology of Maserejian et al. (11) and utilized the food composition table from the open-source database of the Zhejiang University AI and Nutritional Big Data Research Center (https://nutrition.zju.edu.cn).

## Statistical analysis

Statistical analysis was performed using SPSS version 25.0. Continuous data are presented as medians and quartiles. Comparisons between groups of continuous variables with a normal distribution were performed with the *t*-test. Continuous variables without a normal distribution were compared using the Mann‒Whitney *U*-test. A paired *t*-test was used to compare the effects before and after treatment. Comparisons between groups of categorical variables were performed via the chi-square test. Correlation analysis was used to analyse the relationships between sugar intake (including average daily total sugar intake, duration of excessive sugar intake, and average daily sugar intake from the main types of sugary foods) and OAB symptoms (including symptom scores and symptom duration). A *p*-value of <0.05 was considered indicative of statistical significance.

## Results

A total of 97 children diagnosed with OAB were included in this study, with 78 (80.41%) exhibiting excessive sugar intake and 19 (19.59%) exhibiting non-excessive sugar intake. The children were divided into two groups based on their sugar consumption (excessive or non-excessive). Comparisons of clinical data between the excessive sugar intake group and the non-excessive sugar intake group revealed no significant differences in sex, age, BMI, age at which daytime diapers were discontinued, age at which nighttime diapers were discontinued, average urinary flow rate, or maximum urinary flow rate. However, patients in the excessive sugar intake group had a significantly longer duration of OAB symptoms compared to those in the non-excessive sugar intake group (*P* < 0.05) (Table 1).

**Table 1:** Comparison of clinical data of patients with OAB in the excessive sugar intake group and non-excessive sugar intake group.

Items	Excessive sugar intake group	Non-excessive sugar intake group	*P*-value
Average daily total sugar intake (g/day)	71.1 (56.6, 99.2)	6.8 (1.3, 21.7)	0.001
Age (year)	6.5 (5, 9)	6 (4, 8)	0.185
Boys/Girls	59/19	12/7	0.271
BMI	14.9 (13.8, 18.3)	15.2 (12.7, 15.7)	0.400
Duration of OAB symptom (month)	10 (3,17.2)	2 (1, 6)	0.005
Age at which daytime diapers were discontinued (year)	2 (2, 2.5)	2 (2, 2.5)	0.421
Age at which night-time diapers were discontinued (year)	3 (2.5, 3.5)	3 (3, 3)	0.518
Average urinary flow rate (ml/s)	9.4 (7.2, 12.9)	8.2 (6,17)	0.792
Maximum urinary flow rate (ml/s)	13.4 (9.5, 17.6)	10.8 (7.5, 20.1)	0.451

A comparison of OAB symptoms between the two groups revealed that patients in the excessive sugar intake group had significantly higher total scores on the OABSS, as well as higher scores for daytime urination frequency, urgency frequency, and urgency urinary incontinence compared to those in the non-excessive sugar intake group (*P* < 0.05). A comparison of the severity grading of OAB symptoms also revealed that patients in the excessive sugar intake group had more severe OAB symptoms than those in the non-excessive sugar intake group (*P* < 0.05). No significant difference was observed in the number of nocturnal urinations between the two groups (Table 2).

**Table 2:** Comparison of OABss scale score of patients with OAB in the excessive sugar intake group and non-excessive sugar intake group.

Items	Excessive sugar intake group	Non-excessive sugar intake group	*P*-value
Total scores of the OABSS	4 (4, 6)	7 (5, 9)	0.001
Scores of daytime urination frequency	1 (1, 2)	2 (1, 2)	0.016
Scores of urgency frequency	3 (2, 3)	4 (2, 4)	0.021
Scores of urgency urinary incontinence	0 (0, 0)	0 (0, 3)	0.032
Score of the number of nocturnal urinations	0 (0, 1)	0 (0, 1)	0.121
Grade of OAB			
Mild	23 (29.5%)	14 (73.7%)	0.02
Moderate	49 (62.8%)	5 (26.3%)	
Severe	6 (7.7%)	0	

Correlation analysis between sugar intake and OAB symptoms revealed significant positive correlations between average daily total sugar intake and the symptom duration of OAB, OABSS total score, daytime urination frequency score, urgency frequency score, and urgency urinary incontinence score (*P* < 0.05). The duration of excessive sugar intake was significantly positively correlated with the symptom duration of OAB, OABSS total score, and urgency urinary incontinence score (*P* < 0.05). The average daily sugar intake from fruits was also significantly positively correlated with the symptom duration of OAB, OABSS total score, daytime urination frequency score, urgency frequency score, and urgency urinary incontinence score (*P* < 0.05). In contrast, no significant correlations were observed between OAB symptoms and average daily sugar intake from other sources, including carbonated beverages, fruit juices, sugary drinks, sugary tea beverages, chocolate, and ice cream (*P* > 0.05) (Table 3).

**Table 3:** Correlation analysis between sugar intake and OAB symptoms.

Items	Duration of OAB symptom	Total scores of the OABSS	Scores of daytime urination frequency	Scores of urgency frequency	Scores of urgency urinary incontinence	Score of the number of nocturnal urinations
Average daily total sugar intake	*r* = 0.321, *p* = 0.000	*r* = 0.421, *p* = 0.001	*r* = 0.302, *p* = 0.003	*r* = 0.290, *p* = 0.004	*r* = 0.298, *p* = 0.003	*p* = 0.410
Duration of excessive sugar intake	*r* = 0.383, *p* = 0.000	*r* = 0.289, *p* = 0.004	*p* = 0.061	*p* = 0.152	*r* = 0.242, *p* = 0.017	*p* = 0.363
Average daily sugar intake from carbonated beverages	*p* = 0.759	*p* = 0.237	*p* = 0.618	*p* = 0.131	*p* = 0.477	*p* = 0.641
Average daily sugar intake from fruit juices	*p* = 0.237	*p* = 0.072	*p* = 0.215	*p* = 0.082	*p* = 0.449	*p* = 0.521
Average daily sugar intake from fruits	*r* = 0.284, *p* = 0.005	*r* = 0.403, *p* = 0.000	*r* = 0.279, *p* = 0.006	*r* = 0.305, *p* = 0.002	*r* = 0.308, *p* = 0.002	*p* = 0.870
Average daily sugar intake from ugary drinks	*p* = 0.904	*p* = 0.600	*p* = 0.977	*r* = 0.200 *p* = 0.050	*p* = 0.691	*p* = 0.444
Average daily sugar intake from sugary tea beverages	*p* = 0.749	*p* = 0.626	*p* = 0.353	*p* = 0.961	*p* = 0.934	*p* = 0.291
Average daily sugar intake from chocolate	*p* = 0.382	*p* = 0.650	*p* = 0.562	*p* = 0.875	*p* = 0.691	*p* = 0.186
Average daily sugar intake from ice cream	*p* = 0.386	*p* = 0.734	*p* = 0.185	*p* = 0.094	*p* = 0.392	*p* = 0.139

## Discussion

OAB is a common functional bladder disorder in children, with an incidence rate as high as 9.01% among Chinese children. Recent studies reported that obesity, primary nocturnal enuresis, constipation, excessive diaper dependence, bladder–bowel dysfunction, hormonal metabolic disorders, and psychological factors may be associated with the development of OAB (12–14). However, the exact aetiology of OAB remains unclear.

Flores MV et al. (15) reported that insulin resistance and excessive succinate intake are associated with OAB in women with metabolic syndrome. A short-term randomized cohort study by Firouzmand S et al. (16) revealed a direct link between urinary ATP and NO levels and the severity of urinary frequency and urgency symptoms in adult OAB patients. Peyronnet et al. (17) further reported that OAB induced by metabolic syndrome is refractory to traditional anticholinergic therapy and sacral neuromodulation. These studies collectively suggested that high sugar intake leading to metabolic syndrome and insulin resistance can induce OAB in adults and rats. However, these studies have focused primarily on adults, and there is currently a lack of both clinical and basic research data on OAB induced by excessive sugar intake in children. We performed this study to explore the relationship between excessive sugar intake and the occurrence of OAB symptoms in children. Our results revealed that patients in the excessive sugar intake group had significantly higher total OABSS scores, as well as higher scores for daytime urination frequency, urgency frequency, and urgency urinary incontinence compared to those in the non-excessive sugar intake group (*P* < 0.05). Correlation analysis between sugar intake and OAB symptoms revealed significant positive correlations between average daily total sugar intake and symptom duration of OAB, OABSS total score, daytime urination frequency score, urgency frequency score, and urgency urinary incontinence score (*P* < 0.05). The duration of excessive sugar intake was significantly positively correlated with the symptom duration of OAB, OABSS total score, and urgency urinary incontinence score (*P* < 0.05). These findings suggest that, similar to the results of previous studies in adults, excessive sugar intake may also be an important factor contributing to OAB in children.

To further investigate the impact of daily sugar intake from different food sources on OAB symptoms, we found that the average daily sugar intake from fruits was significantly positively correlated with the symptom duration of OAB, OABSS total score, daytime urination frequency score, urgency frequency score, and urgency urinary incontinence score in children with OAB (*P* < 0.05). This association may be related to the effects of fructose (the primary sugar component in fruits) on bladder contractility and stability. In recent years, basic research on the effects of fructose on smooth muscle function has increased. Aimaretti et al. (18) reported that different muscle tissues adapt differently to high-fructose diets and speculated that bladder dysfunction might be associated with mitochondrial dysfunction caused by oxidative stress. Lee et al. (19) recently demonstrated that a high-fructose diet during the postpartum period could induce metabolic syndrome and OAB symptoms in adult mice, with the effects being positively correlated with fructose intake and mediated through specific signalling pathways that affect the relaxation function of the bladder detrusor muscle.

Our study has several limitations. First, this was a single-centre study with a limited number of OAB patients, and the effects of daily sugar intake from some food sources on clinical symptoms may not have reached statistical significance. In particular, the small number of patients with non-excessive sugar intake may introduce bias into the results. Second, the range of food types considered in this study was limited, and potential confounding factors, such as the consumption of small amounts of caffeine in carbonated beverages, may have influenced the results. Third, all participants in this study were patients diagnosed with OAB, and there was no control group of children without OAB. Therefore, while this study can assess the relationship between excessive sugar intake and the severity of OAB symptoms, it cannot be used to determine whether this relationship is related to the occurrence of OAB. In the next phase of the research, we will include a control group to further evaluate the correlation between excessive sugar intake and the development of OAB. Fourth, although children with OAB have lower water intake compared to healthy children, hydration status can influence bladder symptoms. This study did not record the water intake of each child, which may have affected our results. To increase the accuracy and objectivity of future findings, we will include water intake data in future studies.

## Conclusion

Excessive sugar intake was closely associated with the severity of OAB symptoms in children. Both the quantity of sugar intake and the duration of excessive sugar intake were positively correlated with the severity of OAB symptoms. Among all types of sugary foods consumed, the average daily intake of fructose was positively correlated with the severity of OAB symptoms. This study provides a scientific basis for the use of sugar-restricted diets in children with OAB and offers novel insights for the treatment of paediatric OAB.

## Data Availability

The data analyzed in this study is subject to the following licenses/restrictions: The original contributions presented in the study are included in the article, further inquiries can be directed to the corresponding author. Requests to access these datasets should be directed to Xu Cui 16802937@qq.com.
